# Computational Investigation of the Morphology, Efficiency, and Properties of Silver Nano Wires Networks in Transparent Conductive Film

**DOI:** 10.1038/s41598-018-35456-7

**Published:** 2018-11-30

**Authors:** Fei Han, Thirupathi Maloth, Gilles Lubineau, Recep Yaldiz, Amit Tevtia

**Affiliations:** 1 King Abdullah University of Science and Technology (KAUST), Physical Science and Engineering Division, COHMAS Laboratory, Thuwal, 23955-6900 Saudi Arabia; 2 SABIC (Saudi Basic Industries Corporation), P.O. Box 319, 6160 AH Geleen, The Netherlands; 3 SABIC (Saudi Basic Industries Corporation), Thuwal, 23955-6900 Saudi Arabia

## Abstract

Random networks of silver nano wires have been considered for use in transparent conductive films as an alternative to Indium Tin Oxide (ITO), which is unsuitable for flexible devices. However, the random distribution of nano wires makes such conductive films non-uniform. As electrical conductivity is achieved through a percolation process, understanding the scale-dependency of the macroscopic properties (like electrical conductivity) and the exact efficiency of the network (the proportion of nano wires that participate in electrical conduction) is essential for optimizing the design. In this paper, we propose a computational method for identifying the representative volume element (RVE) of nano wire networks. This defines the minimum pixel size in devices using such transparent electrodes. The RVE is used to compute the macroscopic properties of films and to quantify the electrically conducting efficiency of networks. Then, the sheet resistance and transparency of networks are calculated based on the predicted RVEs, in order to analyze the effects of nano wire networks on the electrical and optical properties of conductive films. The results presented in this paper provide insights that help optimizing random nano wire networks in transparent conductive films for achieving better efficiencies.

## Introduction

Optimizing the electro-optical properties of transparent conductive electrodes (TCEs) to satisfy specific applications has long been pursued. TCEs have been widely used in the field of organic photovoltaics (OPVs), organic light emitting diodes (OLEDs), and displays.

There is a trade-off between optical transmissivities (>85%), sheet resistance (<10 Ω/*sq*), haze (>10%), and the production cost to enhance the light absorption and efficiency when using TCEs for photovoltaics^1^. Indium Tin Oxide (ITO) is ubiquitous in transparent and conductive devices due to its ideal combination of excellent sheet resistance (10–100 Ω/sq) and high optical transmittance (>90%). However, energy-intensive processes are involved in manufacturing ITO, and it is too brittle to use in the next-generation flexible electronics^2^. Other oxides such as ZnO:Al (AZO) and SnO2:F (FTO) show similar limitations. Therefore, interest has been shown in researching alternatives^3^.

These alternative materials can be divided into three groups: (1) Carbon-based nanomaterials, such as random networks of single-walled carbon nanotubes^4,5^ and graphene networks^6–8^; (2) Metal nano wire networks, such as random networks of silver^9–13^, copper nano wires^14,15^ or hybrid wires^16^; and (3) Conductive polymers^17,18^. Although the alternative materials have shown some promising results, they either perform worse than ITO or have some drawbacks that hinder them from replacing ITO. Carbon nanotube (CNT), for example, exhibits excellent mechanical and electrical properties, but the high junction resistance at CNT contact points impedes the improvement of the sheet resistance^19^. The high quality of graphene necessary for highly conductive networks is expensive and energy-intensive to produce^20^. Conductive polymers like PEDOT:PSS are electrically unstable as their conductivity decreases when exposed to high temperatures, humidity, or UV light^19^. From among the proposed ITO alternatives, random networks of silver nano wire have been considered the most promising technological and economical compromise so far^1^. Spray-coated silver nanoparticles (Ag-NPs) have been successfully used in inverted solar cells^21^. It is shown that changes in resistance affect the device performance. By increasing the nanoparticle loading, better interconnectivity and morphology is achieved which reduces the sheet resistance and improves the device performance by 3.00%. Spray-coated random silver nano wires (AgNWs) network have also been used to fabricate perovskite solar cells (PSCs)^22,23^. The as-fabricated semi-transparent perovskite solar cell shows an improved photovoltaic output with dual side illuminations due to the transparency of the AgNWs. AgNW layers can surpass commercialized brittle hazy metal oxides and show unique advantages in solar cells applications^24^. In crystalline silicon (Si) solar cells^25^, using random AgNWs networks as transparent top electrodes enhances the conversion efficiencies with respect to conventional metal contact reference cells. The authors claimed the reason was the elimination of shading losses, the preferential scattering of light into the substrate, and the higher charge collection capability with respect to conventional metal contacts. Nevertheless, a number of significant challenges associated with their use remain, most notably optical transmissivities and conductivities of electrode, which are directly affected by nano wires aspect ratio and contact resistance^26^.

The electrical performance of a silver nano wire network is governed by the onset of percolation and the physics of the nano wire contacts. In one computational model of a random nano wire network, the nano wires were modeled in a quasi-2D box with the box height equal to the diameter of a nano wire^27^. Mutiso *et al*.^26^ integrated simulations and experiments to estimate the junction resistance in a nano wire network. They assumed that the junctions dominate the electron transports, neglecting the contribution of the intrinsic nano wire resistances when evaluating the macroscopic sheet resistance. An extension of this work was carried out by Jagota and Nelson^28^, who studied the effect of an anisotropic network orientation on the anisotropy of the sheet resistance. Their simulations showed that the macroscopic sheet conductance in one direction can be improved by restricting the orientation distribution, although conductance in the other direction is lost. Da Rocha *et al*.^29^ went a step further by simulating nano wire networks whose morphology was constructed using data extracted from the images of experimental samples, creating a one-to-one correspondence on the networks between experiments and simulations, and avoiding the statistical averaging that is required for Monte Carlo simulations. They also reported the optimal sheet resistance (assuming zero nano wire-nano wire contact resistance) as a function of the nano wire density.

However, it was not discussed in any of the previous simulations whether the characteristic size of the studied networks is greater than the size of the representative volume element (RVE). The RVE size is the minimum length beyond which the network behaves as a continuous film with respect to some property. It also represents a constraint on the minimum size of electrical devices one can make before sacrificing reliability, and obviously depends on the dimensions of the nano wire and the material coverage. In this paper, the RVE size can be determined based on (1) the geometrical morphology of the random nano wire networks, and (2) the electrical conductivity of the networks. The RVE is defined as the minimum size for which both indicators (morphology and conductivity) become independent on the size of the studied sample. Here, we propose a morphology-based classification and identify the electrical backbone of the network, i.e., the nano wires in the network that carry current, to define the first-approximate RVE size. The RVE size can be determined based on the stabilized fraction of the electrical backbone in the network. After that, we define a more accurate RVE size by estimating the stabilized sheet resistance of the electrical backbone from the former. From an industrial point of view, it is also important to assess the material efficiency, since only a fraction of the nano wires participate in electrical conduction. The efficiency is then a direct measure of the fraction of the nano wires that actually participate to the electrical conductivity.

The paper is organized as follows. In Section 2, the computer generation and classification of a random nano wire network are presented in detail. Then, we describe how to estimate the efficiency of a network, how to determine the RVE size of networks, and how to calculate the sheet resistance and optical transmittance of a network. In Section 3, some numerical examples are evaluated to predict the RVE size, material efficiency, sheet resistance, and transmittance for different network configurations. We end the paper with some concluding remarks in Section 4.

## Computational Models

### Geometric modeling of nano wire networks

**Figure 1:**
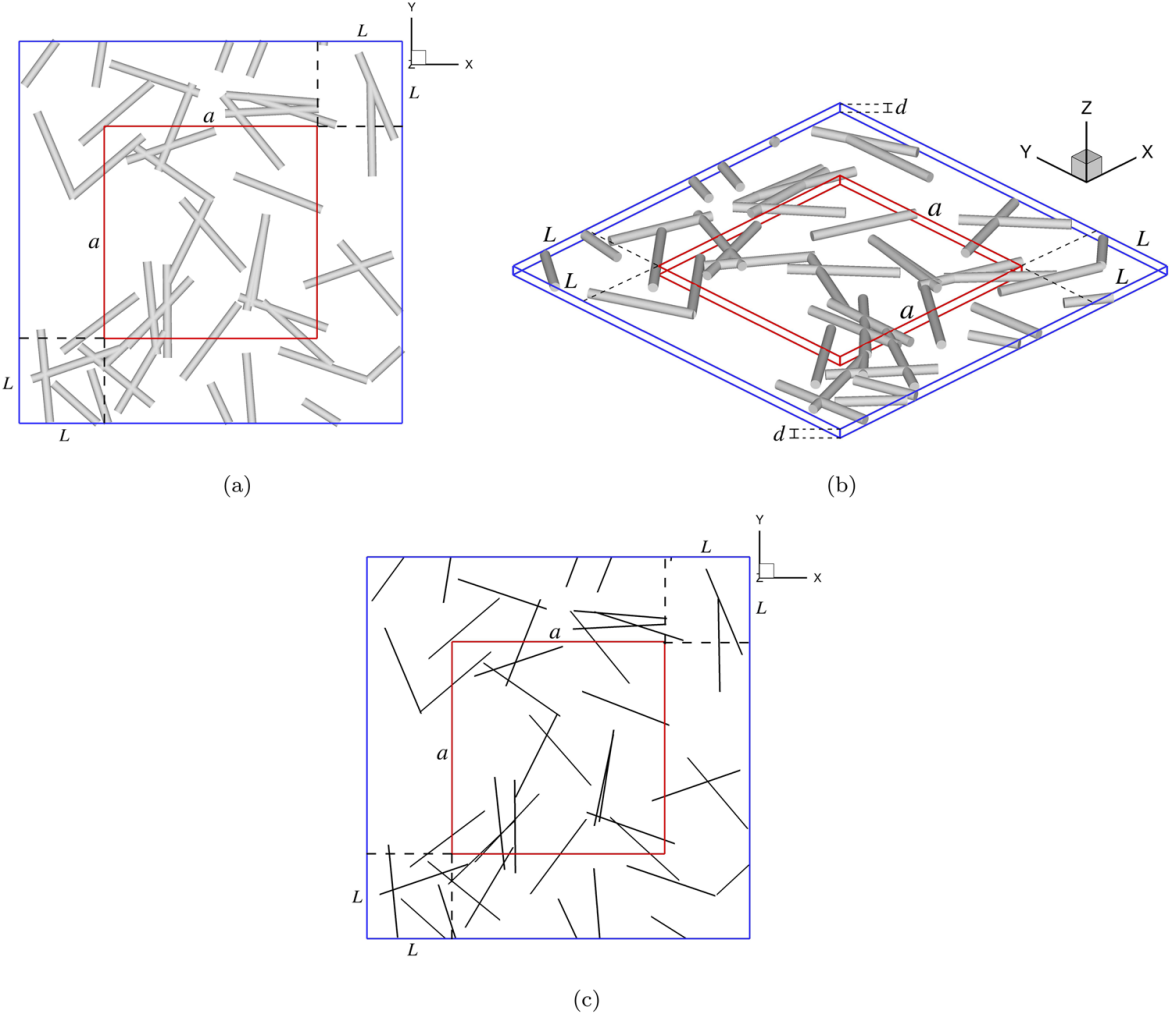
Schematics of the straight and interpenetrable cylindrical rods: (**a**) projection view; (**b**) quasi-2D simulation contained in the cuboid with a height *d*; and (**c**) simplified 2D simulation of straight wires.

A global Cartesian coordinate system is defined by (***x***, ***y***, ***z***). Random networks consisting of straight and interpenetrable cylindrical rods (i.e., right circular cylinders) are generated computationally inside a cuboid with a square base of size [(*a* + 2*L*) × (*a* + 2*L*)] and a height *h*, where *a* is the dimension of the final targeted sample in the ***x*** − ***y*** plane (refer to Fig. 1(a)) and *L* is the length of a rod. A confined 3D structure is achieved by defining the height of the cuboid as *h* = *d*, where *d* is the diameter of the cylindrical rods (refer to Fig. 1(b)). As seen in Fig. 1(b), the schematic of the network shows that the rods are completely confined to the ***z*** direction. Therefore, it is a pseudo-3D configuration called a quasi-2D structure^27^. For simplification, we will describe the steps for generating a 2D straight wire network in the ***x*** − ***y*** plane only (refer to Fig. 1(c)). Then, a complete quasi-2D structure is achieved by considering each wire to be a cylindrical rod with a diameter *d*, as shown in Fig. 1(b).

A straight wire of length *L* is comprised of *n* interconnected segments of length *l* and $n=\lfloor \frac{L}{l}\rfloor +1$, where $\lfloor \bullet \rfloor$ is the greatest integer function. The (*i* + 1)^*th*^ segment of a wire is created between the point *s*_*i*_ and the point *s*_*i*+1_, *i* = 0, 1, 2, …, *n* − 1. The starting point of a wire, *s*_0_, is generated randomly following a uniform distribution in the square region of size [(*a* + *L*) × (*a* + *L*)], i.e., the blue square in Fig. 1. The angle between the straight wire and the ***x*** axis is defined by *θ*, which satisfies a uniform distribution in [0, 2*π*]. Then, the *i*^*th*^ point, *s*_*i*_, of the wire is defined by *s*_*i*_ = *s*_*i*−1_ + *l*(*cosθ*, *sinθ*). Thus, each wire is discretized by *n* + 1 points, rather than by only two endpoints. We discretize the wire by a series of points because: (1) the contact points between two wires can be easily found by a local search, so that the computational cost can be reduced and the parallelization of the generating algorithm can be implemented easily; and (2) this algorithm can be extended to curly wires by changing the orientation of each segment.

Some steps of the algorithm are stated here:

1. To generate *s*_*i*_, *i* = 0, 1, 2, …, *n*, in terms of the computational method stated above;
2. If *s*_*i*_ falls outside of the region of size [(*a* + *L*) × (*a* + *L*)], i.e., outside of the blue square in Fig. 1(c), then we calculate the intersection point of this straight wire with the boundary of the region. Then, we substitute the intersection point for *s*_*i*_, and return to the first step to generate the next point;
3. To calculate the area fraction of wires (considering radius *r*; refer to Fig. 1(a)) within the region of size [*a* × *a*], i.e., the red square in Fig. 1(c), unless both *s*_*i*−1_ and *s*_*i*_ are located outside of the region. If the area fraction is larger than the critical area fraction, *ϕ*_*c*_, then we stop generating points along the wire;
4. If *i* = *n*, then we generate a new wire and return to the first step.

The targeted structure is the network contained in a region of size [*a* × *a*]. The starting point *s*_0_ is generated in an extended region of size [(*a* + *L*) × (*a* + *L*)] in order to minimize the effect of cutting off wires located near or across the region’s boundaries^30^. Thus, this method for generating the targeted region is consistent with the generation of sub-windows within the region during the process of RVE identification (Section 2.3.3).

It is worth noting that the interpenetration among the straight wires is allowed during the network generation. In this paper, the estimated area fraction of wires on the 2D structure (refer to Fig. 1(a)) is defined by *η* = *A*_*s*_/$\bar{A}$, where *A*_*s*_ denotes the sum of the areas of all wires and $\bar{A}$ is the area of the whole region. The real area fraction of the wire network denoted as *ϕ* is the area fraction of the projection of network on the square base. Then, *η* is approximately equal to *ϕ* when *η* is small^31^.

The process mentioned above for generating a quasi-2D structure is a simplified version of the algorithm for a 3D network described in our previous work^32,33^.

### Classification of a network

Not all of the nano wires in a network participate in electrical conduction. In this section, we identify the subset of nano wires in the network that carries current, called the conductive backbone. We go then further by describing the morphology of the networks through a complete analysis of the constitutive clusters, depending on their orientation and on their percolation status. This classification, which describes the overall morphology of a network, is an essential tool for the subsequent RVE and performance determination.

#### Definition of nano wire families

**Figure 2:**
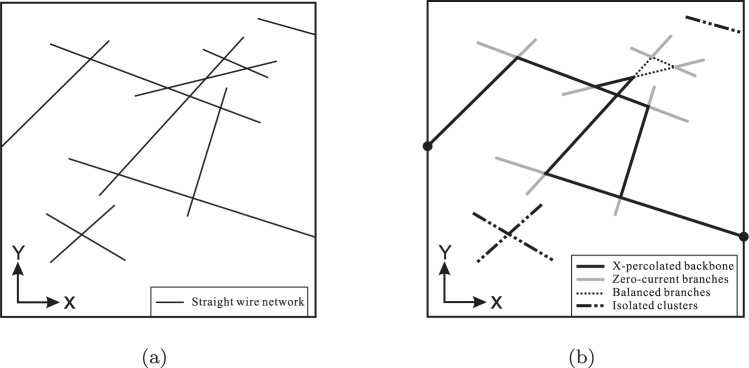
The morphology of a 2D network (**a**), and (**b**) the classification of wires within the network.

We first classify the network into families that are meaningful in terms of the electrical conductivity. A schematic of the network, shown in Fig. 2(a) can be separated into (1) a backbone percolated along the ***x*** direction, (2) some zero-current branches that do not bear any current despite their connection to the percolated cluster, (3) some balanced branches that form closed loops without a current, and (4) some isolated wires or clusters of wires that do not participate in conduction at all because they have no contact with the electrically percolated cluster, shown in Fig. 2(b).

The four types of conductivity found in the network, described above and shown in Fig. 2, are classified into the four families given below:

1. X-percolated backbone: the backbone structure percolated only along the ***x*** direction (area fraction: *f*_X_),
2. Y-percolated backbone: the backbone structure percolated only along the ***y*** direction (area fraction: *f*_Y_),
3. XY-percolated clusters: the backbone structure percolated along both the ***x*** and ***y*** directions (area fraction: *f*_XY_), and
4. Nonpercolated (NP) clusters/wires: isolated wires, zero-current branches, and balanced branches (area fraction: *f*_NP_).

Using this classification, each segment can only belong to one of these families (i.e., *f*_X_ + *f*_Y_ + *f*_XY_ + *f*_NP_ = 1 and *F*_*i*_ ∩ *F*_*j*_ = ∅, where *F*_*i*_ and *F*_*j*_ are any of the families defined above and *i*, *j* = X, Y, XY, and NP).

#### Identification of geometrically percolated structures

We utilize the Hoshen Kopelmann algorithm^34,35^ to identify wire clusters by finding the contact points between any two wires. We say that two points have contact with each other when they are separated by a distance less than or equal to the wire diameter, *d*.

**Figure 3:**
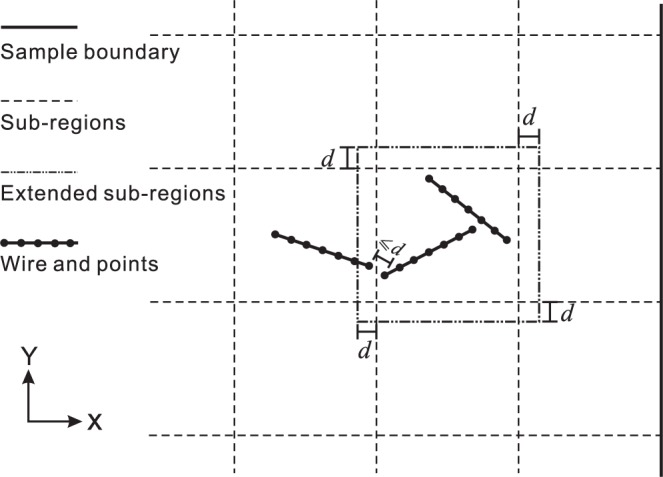
2D schematic of the sub-regions and an extended sub-region.

However, it is computationally expensive to find all of the contact points within a dense network because of the large number it may contain. Therefore, a sample square of size [*a* × *a*] is divided into *k* segments along each direction, resulting in *k*^2^ sub-regions. Then, each sub-region is searched for contact points, instead of the whole sample. Two contact points may belong to different sub-regions when they are close to the sub-region boundaries. To take these points into account, each sub-region is extended externally by the diameter distance, *d*, along each direction, ***x*** and ***y***, as shown in Fig. 3, since the maximum distance between two contacting points is equal to *d*.

The four boundaries of the square region are labeled *x*_0_, *x*_1_, *y*_0_, and *y*_1_. If a cluster contains wires in contact with the *x*_0_ (respectively, *y*_0_) and *x*_1_ (respectively, *y*_1_) boundaries, then we say that this cluster percolates in the ***x*** (respectively, ***y***) direction, i.e., it is an X- (respectively, Y-) percolated cluster. If a cluster contains wires that are in contact with all of the boundaries, *x*_0_, *x*_1_, *y*_0_, and *y*_1_, then it is an XY-percolated cluster. The clusters that do not percolate at all are NP clusters.

#### Extraction of electrically conductive backbones

**Figure 4:**
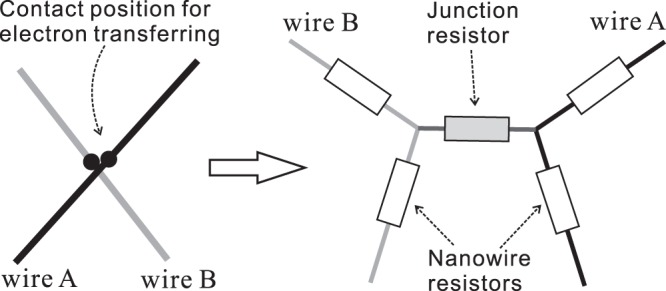
On the left, two wires, denoted by A and B, cross each other to form an electron transfer pathway near their discretized points. On the right, the corresponding network of resistors including nano wire resistors and a junction resistor is shown.

From the geometrically percolated network obtained above, we extract the electrically conductive backbone by removing the non-conducting branches. Here, the direct electrifying algorithm^36,37^ is applied to determine the zero-current pathways, i.e., the non-conducting branches. We first convert the geometrically percolated network into a resistor system. In this system, two types of resistors are considered: (1) the nano wire resistor and (2) the junction resistor between two contacting points. The junction resistor is created by the contact resistance between surfaces of two silver nano wires. When a junction resistor is added between two wires, each of the wires is divided into two resistors, as illustrated in Fig. 4. To simplify the computation, we assign 1 Ω to each resistor in the network. Then, a voltage is applied to the opposite boundaries of the whole sample. This voltage is set to be ten times the number of points, so that the difference between the non-zero and zero current values is easily distinguished through numerical solutions^33^. Then, we calculate the current passing through each resistor by solving the equations of the resistor network obtained through Kirchhoff’s current law. If the current passing through a resistor is non-zero, then that resistor corresponds to a wire in the electrically conductive backbone. On the other hand, if a resistor does not carry any current, then it corresponds to a non-conductive branch.

### Estimation of network performances

#### Utilization efficiency of nano wires in a network

The backbone structure of a network plays the main role in conducting electricity. Therefore, we define the utilization efficiency of nano wires in a network as the fraction (*f*_X_ + *f*_Y_ + *f*_XY_) of its conductive backbone. This “backbone fraction” becomes an index that indirectly reflects the electrical conductivity of a network. In this way, the conductive efficiency of a network with given dimensions can be accurately quantified by the backbone fraction.

#### Electrical conductivity of a network

For a given conductive network consisting of silver nano wires, the sheet resistance is mainly a result of its conductive backbone. Here, the direct electrifying algorithm^36,37^ is used again to calculate the electrical resistance of the backbone structure. As described in Section 2.2.3, the backbone structure is converted into a resistor system consisting of the nano wire resistors and the junction resistors. The resistance of a nano wire resistor can be estimated by the geometry of the nano wire segment and the resistivity of the silver. The junction resistance can be an arbitrary, reasonable value, which we assume to be constant for all the junctions in the network in our simulations. Some post-processing methods, like thermal annealing^38^, mechanical pressure^12^, chemical welding^39^, and electrowelding^40,41^, have been used to dramatically reduce the junction resistance by welding two contacting nano wires together. Hence, the extreme sheet resistance of a given network comes entirely from the skeleton of the network when the junction resistance is equal to zero.

After we establish the resistor system, voltage can be applied to the backbone network of resistors. The electrical equations that define the voltage distribution over the resistor system are solved based on Kirchhoff’s current law. Then, the total current through the system is estimated by using Ohm’s law on each resistor. Finally, the equivalent resistance of the network is evaluated.

#### Determination of the RVE size

An RVE containing wires in two dimensions is considered to be the smallest region over which a simulation result will yield values of an effective property, such as the backbone fraction or sheet resistance, they are representative of the whole sample.

**Figure 5:**
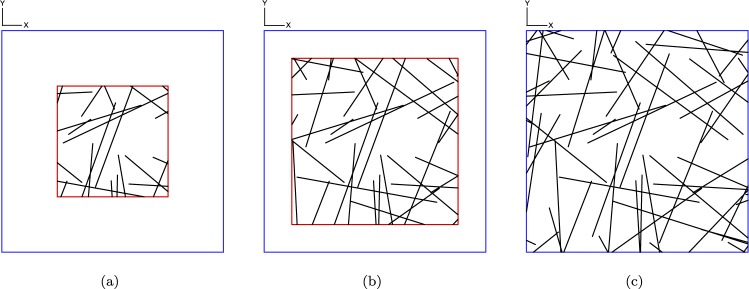
Schematics of the process for finding the critical size of an RVE. The size of the blue square is *a* = 100 *μ*m. The size of the red square in (**a**) is *b* = 50 *μ*m and in (**b**) is *b* = 75 *μ*m; Δ*b* = 25 *μ*m.

To estimate the RVE size, we start by defining an observation window of size [*b* × *b*] inside a global region of size [*a* × *a*], where $b\le a$. Here, we assume that the region of size [*a* × *a*] is large enough to contain an RVE of the network. The observation window remains at the center of the region even as the size of the window increases. The size of the observation window is progressively increased in increments of Δ*b* until it reaches the size of the global region, i.e., until *a* = *b*. Schematics of the process are shown in Fig. 5.

For each observation window, we extract the electrically conductive backbone of the network and then calculate the fraction of each family defined in Section 2.2.1, i.e., *f*_*i*_, where *i* denotes X, Y, XY, or NP. At the same time, we calculate the sheet resistance of the network using the conductive backbone in the observation window. Finally, both the backbone fraction and the sheet resistance are analyzed, as the size of observation window increases, in order to find the size of the RVE. It is worthy to note that the RVE sizes determined by the backbone fraction and the sheet resistance are different.

#### Optical transmittance

The optical transmittance of a silver nano wire network stems from the open spaces in the network where light can propagate. Naturally, the transmittance deteriorates when the nano wire network is very dense. Experiments and simulations suggest that the optical transmittance is a strong function of the nano wire diameters. In conjunction with the optimal electrical properties predicted, a simple empirical relation for finding the optical transmittance is applied when the wavelength of light is set to 550 nm^31^:

1 $$ \% T=100-{a}_{1}\times \eta ,$$

where *a*_1_ is a fitting parameter which is calculated as

2 $${a}_{1}=100\times \,\mathrm{ln}(10)\times {Q}_{ext},$$

where *Q*_*ext*_ is the dimensionless extinction efficiency of the nano wires and a function of the nano wire diameter. For example, if the nano wires are 30 nm (respectively, 300 nm) in diameter at 550 nm wavelength, then *Q*_*ext*_ yields 0.28 (respectively, 2.1)^42^ and, hence, *a*_1_ = 64.5 (respectively, *a*_1_ = 483.5) by Eq. (2). It is worthy noting that Eq. (1) holds only when *η* approaches zero because the first order Taylor approximation is used in its derivation^31^.

In the following, we also intend to clarify how optimal properties can be obtained based on the utilization of random networks only. It is well known that, for a given area of fraction of silver of a transparent substrate, the optimal configuration to get the best isotropic conductivity is to have a perfect square-grid arrangement of the conducting material. Of course, the efficiency of such a square grid results from (1) an optimal microstructure without disorder in which all material participates to the conduction (2) the fact that there is no contact resistance in such a monolithic grid. To see how well random networks can compete with this optimal configuration, we will assume random networks without contact resistance. The contact resistance can indeed be largely decreased by using some welding processes that were introduced recently^12,38–41^. So we compare the optical transmittance and sheet resistance of an ideal (i.e., the junction resistance vanishes) random network to those of a regular grid^43^. This is important from a practical point of view. Indeed, despite they are an optimal configuration, regular square grids are relatively more difficult to manufacture. Random networks can be very easily obtained by spray deposition or other techniques^44–48^.

## Numerical Results

In this section, networks consisting of randomly oriented nano wires are studied numerically. The statistical results of 25 samples are used to find the RVE size and to estimate the efficiency of the networks; 10 samples are used to study the conductivity and transparency of the networks. The electrical resistivity of silver is set to 2.26 × 10^−8^ Ω m^29^, and the nano wire diameter is 50 nm in all of the examples. We consider nano wires of four distinct aspect ratios, 50, 100, 300, and 600, for different networks, in order to study their effects on the networks. The lengths of the nano wires can be calculated from the diameter and the aspect ratios (given in Table 1). The diameter and lengths of nano wires given here are typical commonly used silver nano wires^49^. The critical area fractions of the percolated networks, i.e., the percolation thresholds *η*_*c*_, are obtained from the Fig. 2 in^27^ and are provided for reference in Table 1. The area fractions *η* applied in all of the simulations are within the range [1.3415*η*_*c*_,4.981*η*_*c*_], which includes area fractions both near to and far from the percolation threshold.

**Table 1:** Percolation threshold (*η*_*c*_) for nano wire networks with different aspect ratios and lengths of nano wires.

Aspect ratio	nano wire length	*η*_*c*_
50	2.5 *μ*m	0.1068
100	5 *μ*m	0.0552
300	15 *μ*m	0.019
600	30 *μ*m	0.009621

### RVE size of networks

Next, we will demonstrate how to find the RVE size of networks in terms of the backbone fraction and the sheet resistance. Then, we compare the RVE sizes obtained by the backbone fraction with those obtained by the sheet resistance.

#### RVE size based on backbone fraction

**Figure 6:**
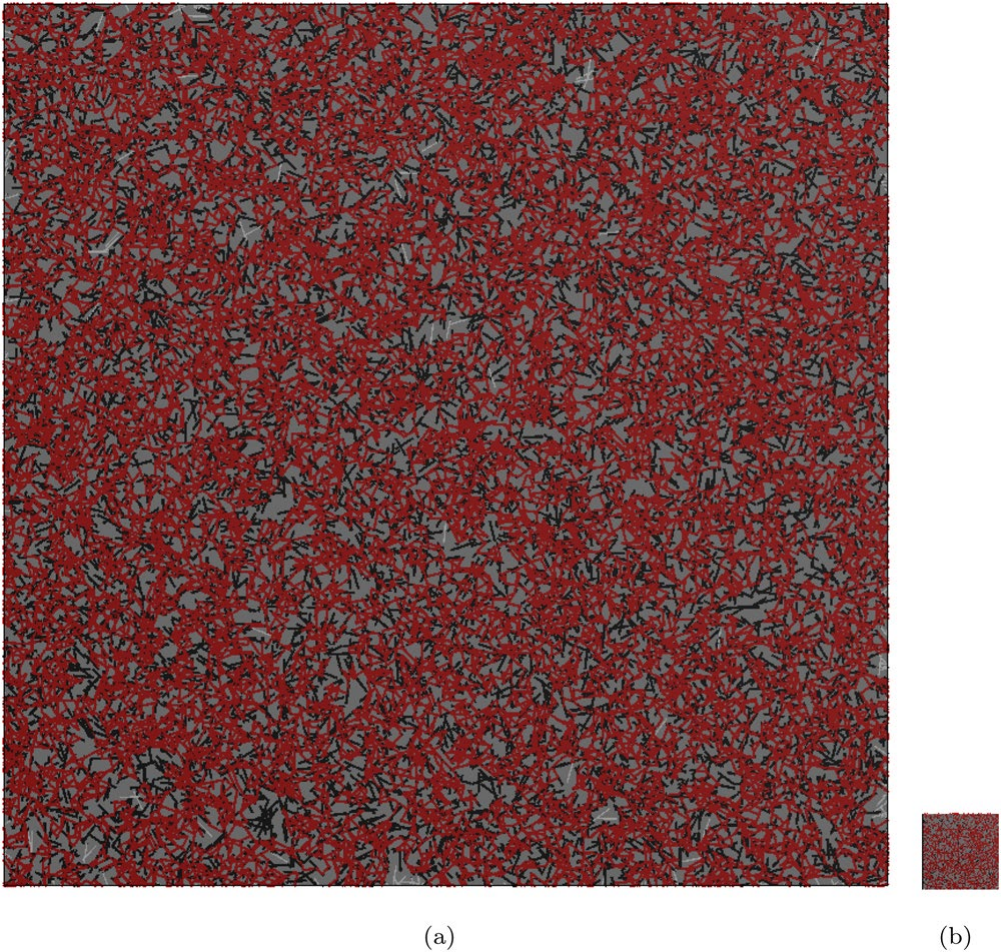
Images of networks with different area fractions but the same nano wire aspect ratio (i.e., 50): (**a**) *η*/*η*_*c*_ = 1.631, *S*/*L* = 46.54; (**b**) *η*/*η*_*c*_ = 4.981, *S*/*L* = 3.992, where the normalized window size is defined as *S*/*L*; *S* denotes the size of windows and *L* is the length of nano wires. XY-percolated backbone is red, zero-current and balanced branches are black, and the few isolated nano wires are white.

We show the morphologies of 2D networks with different area fractions in Fig. 6. nano wires with the aspect ratio 50 are used to generate the networks shown in both Fig. 6(a,b). According to Table 1, the percolation threshold, *η*_*c*_, is 10.68%. For a smaller area fraction of percolated networks, the percolated network is needed to generated in a window with the larger normalized size (*S*/*L*, see Fig. 6). On the other hand, a network with the larger area fraction only needs a window with the smaller normalized size to obtain stable predicted results. For example, when *η*/*η*_*c*_ = 1.631, the normalized size is *S*/*L* = 46.54 (Fig. 6(a)); When *η*/*η*_*c*_ = 4.981, the normalized size is *S*/*L* = 3.992 (Fig. 6(b)).

**Figure 7:**
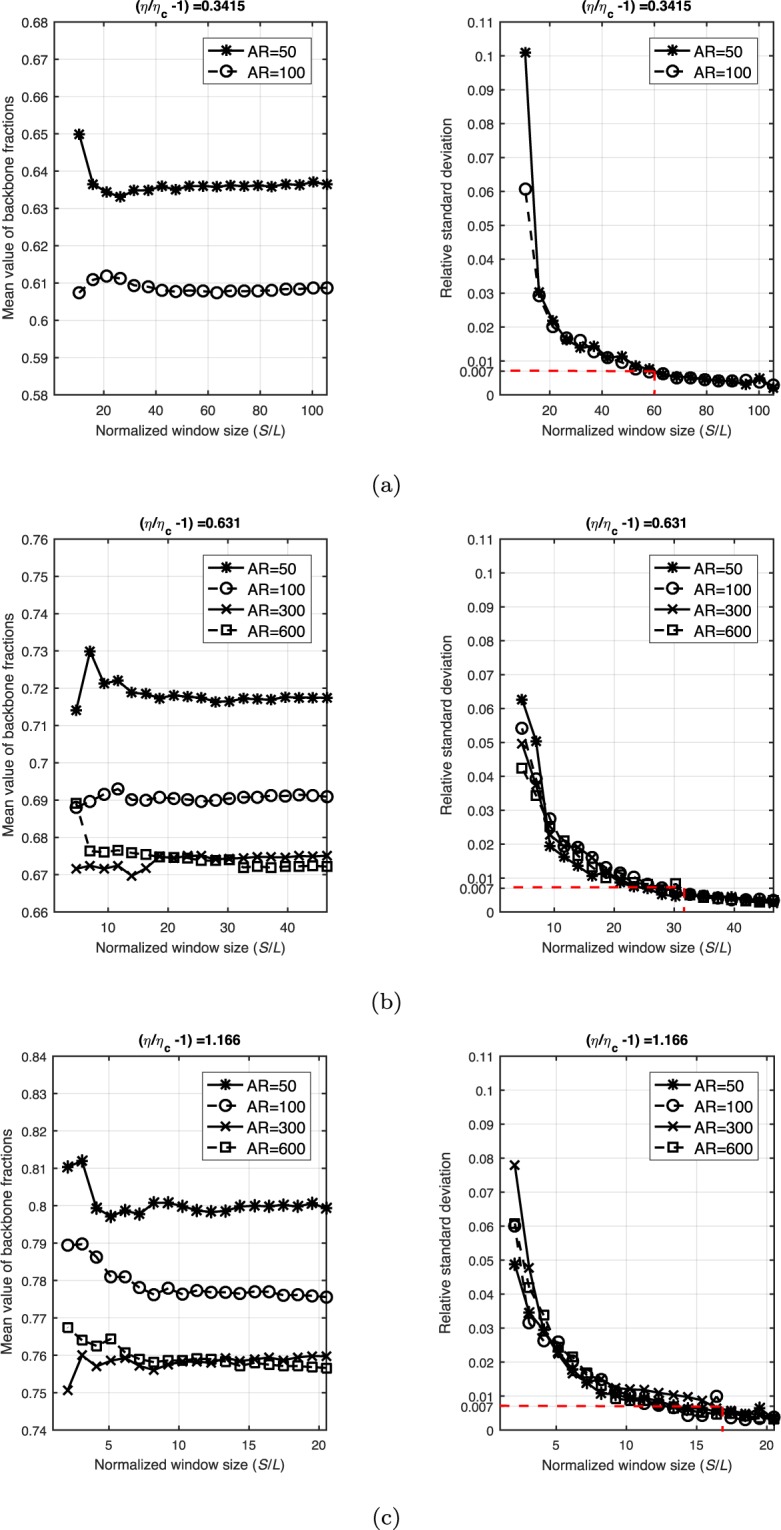
Stabilization of backbone fractions with normalized window sizes (*S*/*L*: *S* denotes the size of windows and *L* is the length of nano wires) is shown by the mean value (in the left column) and the relative standard deviation (in the right column) of backbone fractions. The red dashed lines in the right column indicate the estimated RVE sizes.

Figure 7 shows the variation in backbone fractions with the normalized observation windows. Different effective filling factors, which is defined by *η*/*η*_*c*_ − 1, are shown from Fig. 7(a–c). As defined before, the RVE size (indicated in Fig. 7 by the red dashed lines) is the smallest length above which fluctuations (i.e., relative standard deviation) in the backbone fraction decline slowly and all fall below a small threshold (0.007 in this example).

#### RVE size based on sheet resistance

**Figure 8:**
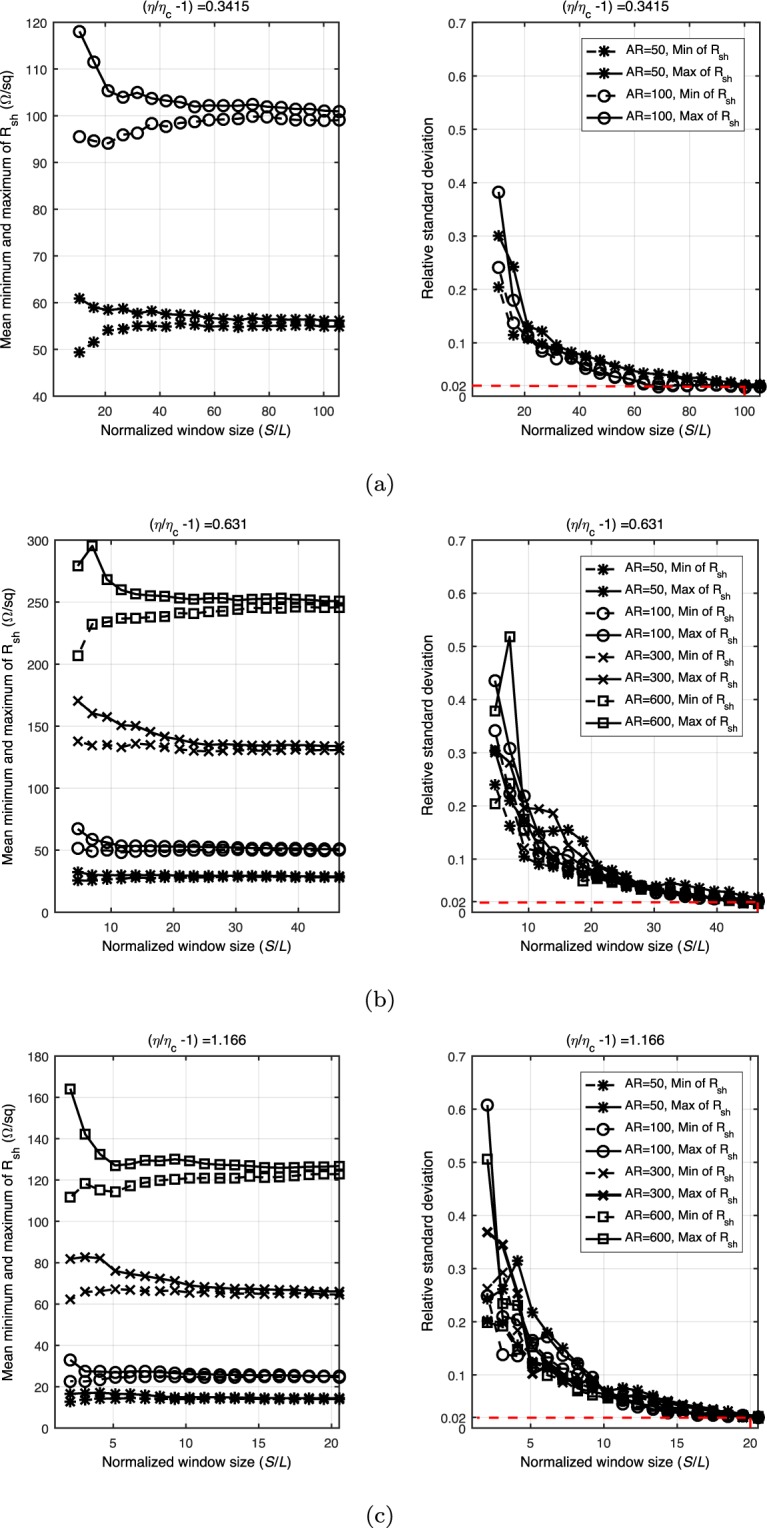
Stabilization of sheet resistances with normalized window sizes (*S*/*L*: *S* denotes the size of windows and *L* is the length of nano wires) is shown by the mean minimum and maximum values (in the left column) and the relative standard deviation (in the right column) of sheet resistances. The red dashed lines in the right column indicate the estimated RVE sizes.

After we determine the electrical backbone of samples, the sheet resistance of the backbone is calculated. In these simulations, the junction resistance is considered constant at 10 Ω for all contacts between the nano wires^50^. For a given sample, we calculate its sheet resistances along the ***x*** and ***y*** directions. The sheet resistance components are different about the ***x*** and ***y*** directions due to the random distribution of the nano wires. Then, we compute the mean minimum and mean maximum sheet resistances from all of samples of each window size and aspect ratio of the nano wires. The left column of Fig. 8 shows the mean minimum and mean maximum values of each window size for different area fractions. These results indicate that the sheet resistance is anisotropic when the normalized window size is small, but becomes isotropic when the window size increases. The fluctuations in both the minimum and maximum resistances are measured by the relative standard deviation for different aspect ratios, and are shown in the right column of Fig. 8. From the sub-figures, we see that the relative standard deviations decline slowly as they approach the critical value of 0.02. We choose 0.02 as the threshold for two reasons: (1) the sheet resistances of the networks become isotropic, and (2) for the given area fraction of the networks, the fluctuations are independent of the aspect ratios. Consequently, the corresponding window size is identified to be the size of the RVE based on the sheet resistance.

**Figure 9:**
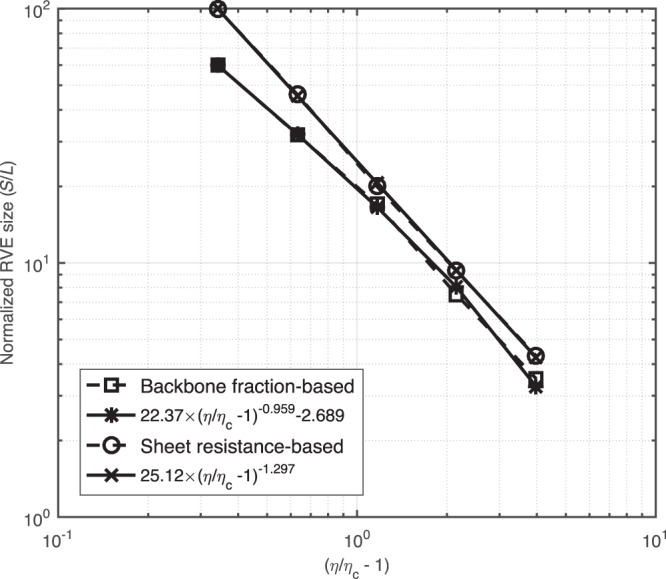
Comparison and fitting of the RVE sizes identified by the backbone fraction and the sheet resistance.

We plot the two RVE sizes, based on the backbone fraction and based on the sheet resistance, in Fig. 9 as functions of the effective filling factors, i.e., *η*/*η*_*c*_ − 1, then fit and plot a power function for each RVE size. The results suggest that if the area fraction is small and close to the percolation threshold, then a sample size of at least 100 times the length of a nano wire is required to make a meaningful electrical measurement on an RVE. When the increased area fraction is much greater than the percolation threshold, a sample size of 1∼10 times the length of a nano wire is required to make electrical measurements. Here, the identified RVE size also indicates the minimum size of a conductive film that one can be fabricated with a reliable electrical performance.

It is clear from Fig. 9 that the RVE size based on the sheet resistance is larger than the RVE size based on the backbone fraction even though a greater relative standard deviation (i.e., 0.02) is applied in our simulations, which implies that if one wants to make meaningful electrical measurements, then the size should be based on the sheet resistance in order to guarantee stable morphological and electrical properties. In our simulations, the sheet resistance is calculated based on the backbone structure. Thus, the RVE size results based on sheet resistances incorporate the effect of the backbone. For example, the anisotropic sheet resistances reflect the nonuniform configuration of the backbone structure. Until the predicted results become isotropic, the corresponding window with an increased size can be recognized as the RVE, which represents the uniform network on the macroscale.

### Efficiency and conductivity of a network

**Figure 10:**
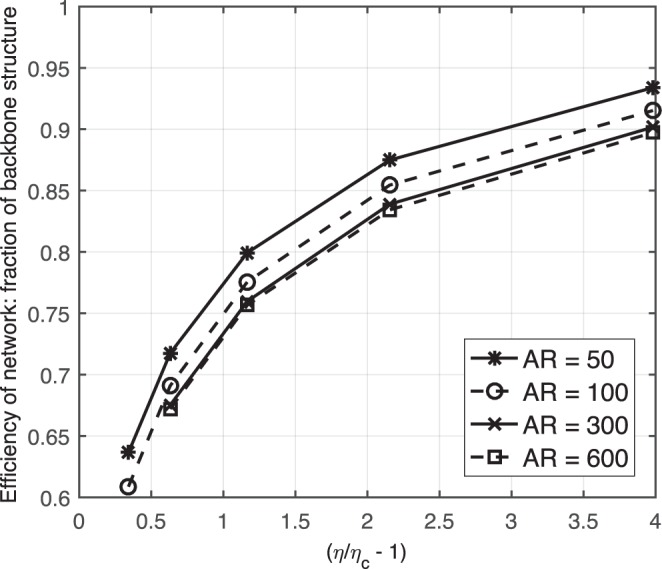
Efficiencies of networks as measured by the electrical backbone fraction with different aspect ratios of nano wires.

According to the above simulation results, we plot the efficiency of each network, in terms of the fraction of the electrical backbone in the network, as a function of the effective filling factor, i.e., *η*/*η*_*c*_ − 1 (refer to Fig. 10). Two key points can be observed from Fig. 10:

1. When the area fraction is close to the percolation threshold, more than 30–40% of the nano wires in a network fail to participate in the electron transport. When the area fraction of a network increases greatly, most of the network becomes conductive.
2. At the same effective filling factor, the efficiency is higher with nano wires that have a low aspect ratio than a high aspect ratio for the following reasons. There are more nano wires with a low aspect ratio than with a high aspect ratio when the area fraction of nano wires is the same. Therefore, for the same diameter, Shorter nano wires make a more uniform distribution, which leads to the shorter nano wires having more opportunities to connect with each other.

**Figure 11:**
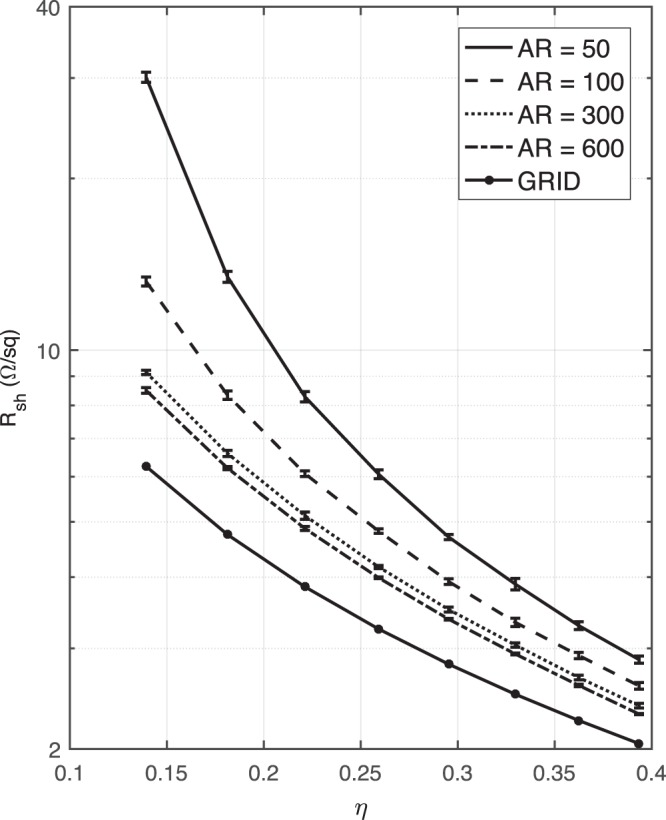
The sheet resistances of ideal random networks (i.e., the junction resistance *R*_*j*_ = 0 Ω) along with error bars as a function of area fractions, as compared to the grids with the same area fractions.

Applications usually target the smallest sheet resistance possible. One way is to reduce the junction resistance through post-processing^12,38–41^. Another way is to understand the effects of the area fraction and aspect ratio on the sheet resistance. In this work, we focus on the latter. Therefore, we choose a perfect junction resistance, i.e., *R*_*j*_ = 0 Ω, in our simulations. In addition, the sheet resistance of the regular grid is estimated^43^ as this is known to represent the best possible performance for a given coverage. Then, we plot the sheet resistance as a function of the area fractions. As can be seen in Fig. 11, a higher aspect ratio leads to a lower sheet resistance because the longer nano wires connect more easily and create shorter conductive pathways. When the area fraction increases, the difference between sheet resistances of higher and lower aspect ratios decreases, due to the shorter nano wires producing a more uniform distribution.

### Optimal transparency and conductivity

For transparent conductors, both the sheet resistance and the optical transmittance are primary parameters for applications. A transparent conductive film must exhibit both a low sheet resistance (R_sh_ < 10 Ω/sq) and a high optical transparency (%*T* > 80%). A common way to quantify the performance of the conductive film is to introduce a figure of merit (FoM). Such FoM can be used to compare a technology to known standards. The transmittance can be related to the sheet resistance:

3 $$T={(1+\frac{{Z}_{0}}{2{R}_{s}}\frac{{\sigma }_{op}}{{\sigma }_{DC,B}})}^{-2}$$

**Figure 12:**
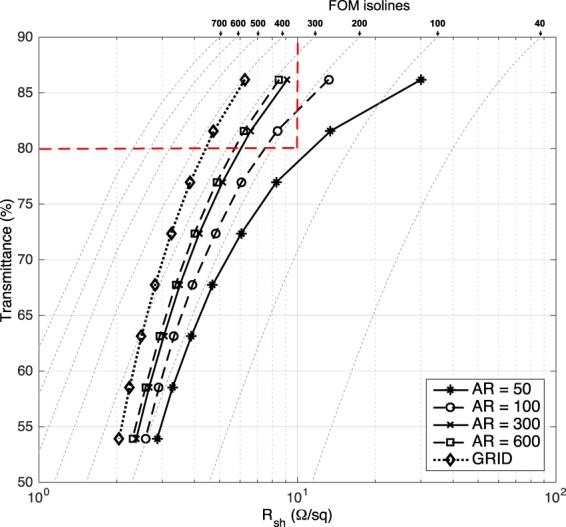
The transparency and resistance of ideal random networks with different aspect ratios of nano wires, as compared to the grid networks with the same area fractions. Isolines correspond to different values of the FoM as defined in section 3.3.

*Z*_0_ is the impedance of free space (377 Ω). A natural dimensionless FoM is then defined as the ratio of the DC to optical conductivity: *σ*_*op*_/*σ*_*DC*,*B*_^51^. The isolines of this FoM are plotted on Fig. 12, for isovalues ranging from 40 (which already corresponds to a film with acceptable performances) to 700 (which corresponds to the best values reported so far^24^).

Figure 12 is the calculated plot of optical transmittance vs. sheet resistance, with different aspect ratios of nano wires and regular grids. The corresponding relationship between the sheet resistance and the area fraction are plotted in Fig. 11. A regular grid is the ideal configuration to achieve isotropic sheet resistance and high optical transmittance^43^. In the simulations, the junction of nano wires is still *R*_*j*_ = 0 Ω. The transmittance is calculated from Equation (1), and the fitting parameter is chosen to be *a*1 = 100 × ln(10) × 0.4 ≈ 92.1 (refer to Equation (2)) in our simulations, where the extinction efficiency *Q*_*ext*_ = 0.4. It has been reported that *Q*_*ext*_ = 0.28 for a nano wire 30 nm in diameter and *Q*_*ext*_ = 2.1 for a nano wire 300 nm in diameter^42^. If we refer to above values and assume that *Q*_*ext*_ is a linear function of diameter, then we can directly estimate that *Q*_*ext*_ ≈ 0.4 for a nano wire 50 nm in diameter. Figure 12 shows that only the regular grid and random networks with aspect ratios higher than 100 and larger area fractions achieve the transmittance and sheet resistance (i.e., are within the red dashed zone of Fig. 12) required to produce display devices.

## Conclusions

A computational method has been developed to investigate the morphology, utilization efficiency, and electrical properties of a silver nano wire network. The size of an RVE for a nano wire network is determined by analyzing the stabilization of the backbone fractions or the sheet resistances in a series of observation windows with different sizes. When the concentration of nano wires is low and near the percolation threshold, the size of an RVE is 50∼100 times the length of the nano wires; when the concentration of nano wires is high and far from the percolation threshold, the size of an RVE is only 3∼6 times the length of the nano wires. For the utilization efficiency of nano wires in a sparse network, we have found from our simulations that only 60∼70% of the nano wires participate in electrical conduction, due to the randomness of the nano wire dispersion.

To improve the conductivity of a silver nano wire network, one may consider increasing the concentration of nano wires. However, this method decreases the optical transmittance of the network. High optical transmittance is also a major requirement of transparent conductive film. Consequently, we recommend improving the utilization efficiency of nano wires in a random network by increasing both the aspect ratio and the concentration of the nano wires, to achieve better optical and electrical properties.
